# Ethics of animal research in human disease remediation, its institutional teaching; and alternatives to animal experimentation

**DOI:** 10.1002/prp2.332

**Published:** 2017-08-09

**Authors:** Rajkumar Cheluvappa, Paul Scowen, Rajaraman Eri

**Affiliations:** ^1^ Department of Medicine St. George Clinical School University of New South Wales Sydney New South Wales Australia; ^2^ Department of Animal Services University of Tasmania Hobart Tasmania Australia; ^3^ Mucosal Biology Laboratory School of Health Sciences University of Tasmania Launceston Tasmania Australia

**Keywords:** alternatives, animal ethics committee, animal experimentation, animal research, code, distress, ethics, pain, pathophysiology, reduction, replacement

## Abstract

Animals have been used in research and teaching for a long time. However, clear ethical guidelines and pertinent legislation were instated only in the past few decades, even in developed countries with Judeo‐Christian ethical roots. We ***compactly*** cover the basics of animal research ethics, ethical reviewing and compliance guidelines for animal experimentation across the developed world, “our” fundamentals of institutional animal research ethics teaching, and emerging alternatives to animal research. This treatise was meticulously constructed for scientists interested/involved in animal research. Herein, we discuss key animal ethics principles – Replacement/Reduction/Refinement. Despite similar undergirding principles across developed countries, ethical reviewing and compliance guidelines for animal experimentation vary. The chronology and evolution of mandatory institutional ethical reviewing of animal experimentation (in its pioneering nations) are summarised. This is followed by a concise rendition of the fundamentals of teaching animal research ethics in institutions. With the advent of newer methodologies in human cell‐culturing, novel/emerging methods aim to minimise, if not avoid the usage of animals in experimentation. Relevant to this, we discuss key extant/emerging alternatives to animal use in research; including organs on chips, human‐derived three‐dimensional tissue models, human blood derivates, microdosing, and computer modelling of various hues.

**Abbreviations**
ECPA: European Crop Protection Association
EFPIA: European Federation of Pharmaceutical Industries and Associations
HSE: human skin equivalents
ICLAS: International Council for Laboratory Animal Science
SPCA: Society for Prevention of Cruelty to Animals

## Introduction

The humanest possible treatment of experimental animals, far from being an obstacle, is actually a prerequisite for successful animal experiments.

— Russell & Burch. Principles of Humane Experimental Technique (1959)(Russell and Burch 1959).

The use of animals in pathology, and related research/teaching is pivotal to the advancement of science, as animals have been considered to be good model systems for humans and human disease. Animal models can be appropriate, or can be approximated to study human anatomy, physiology, pathology, etc., as animals may have a biological milieu resembling human homoeostatic conditions.

Research involving animals may include awareness research (e.g., behavioural, embryological, physiology, and genetic) which is necessary to contribute eventually (and indirectly) to human disease remediation (Cheluvappa et al. 2007a,b), and applied research (academic or/and commercial), such as pathology (Cheluvappa et al. 2015), drug testing, pathogen research (Cheluvappa et al. 2010), defence research, and toxicology (Cheluvappa et al. 2008). When animal research ethics are mentioned subsequently in this work, it will generally refer to the academic areas which we (the authors) work with, namely; murine models of physiology, pathogenesis, and toxicology.

Animal usage in research and teaching is subject to strict ethical guidelines all over the developed world. With the advancement of technology in medical research, we are now at a stage to consider manifold alternatives to utilising animals in research and teaching. In this study, we ***concisely*** cover the fundamental principles of animal research ethics, compliance guidelines for animal experimentation, institutional animal research ethics teaching, and emerging alternatives to animal research. The intended targets of this study are scientists and ethicists interested in animal research, for example, undergraduates, graduate students, medical students, and clinicians. We emphasise that this work is ***not*** intended to be an elaborate treatise.

The intents of this study are fourfold, with each intent in contiguity with the next.

1. The first intent of our study is to provide a concise summary of previous and extant thought on animal experimentation ethics.
2. The second intent of our study is to demonstrate how previous and contemporary thought on principles/mores pertaining to animal experimentation ethics, finally translated into concrete legislation to mandate compulsory review of ethical practices in animal research.
3. The third intent of our study is to lay out suggestions, practical recommendations, and teaching strategies for the lucid inculcation of animal experimentation ethics to interested parties.
4. The fourth intent of our study is to provide and raise awareness of available alternatives to animal research.

## Ethics in animal experimentation – the beginnings and the basics

The first intent of our study is to provide a summary of historical and extant thought on animal experimentation ethics in human disease elucidation and therapy, inclusive of extant thought that is more or less accepted around the developed world. This intent also extends an implicit encouragement to relevant personnel to conform to these ethical principles and standards.

Animal ethics are not stringent rules mandating researchers to conduct animal research in certain ways, but an arena for promoting the expression of human moral obligations towards animals used in research. However, Russell and Burch (1959) set of 3Rs (Replacement, Reduction, and Refinement) is arguably the best known, and the most utilised set of animal ethics to date.

Despite Greek and Roman references to animal experimentation by Aristotle (4th century BC) (Cohen and Loew 1984), Erasistratus (3rd century BC)(Cohen and Loew 1984), and Galen (2nd century AD)(Greek and Greek 2000), the earliest reference to animal welfare and ethics occurs only in the 19th century (Zurlo et al. 1994). For example, what we know as Society for Prevention of Cruelty to Animals (SPCA) today, was originally organised in England in 1822 (Zurlo et al. 1994). In 1831, the first seeds were sown for today's animal ethics guidelines by Marshall Hall, a British physiologist (Zurlo et al. 1994). In 1876, the English House of Commons passed the first bill relating to animal experimentation (the Cruelty to Animals Act 1876 = An Act to Amend the Law Relating to Cruelty to Animals 1876) following which, a number of countries including USA followed suit (Zurlo et al. 1994). In 1959, William Russell, an intelligent young zoologist (then), psychologist and scholar; and Rex Burch, a microbiologist, published “The Principles of Humane Experimental Technique.” Therein, they categorised humane animal experimentation techniques (Part 2 The Progress of Humane Technique) under replacement, reduction, and refinement, now referred to as the 3Rs – Replacement, Reduction, and Refinement (Russell and Burch 1959).

Animal experimentation ethics did not emerge de novo. It evolved over centuries of philosophical traditions. Concepts such as Aristotle's virtue ethics (Cohen and Loew 1984) (ethical treatment of animals stem from the “character” of individual humans), Hobbe's 17th century contractarianism (Rowlands 2013) (acceptable if most people accept the experimental objectives without offence), Kant's (1788) deontological approach (beneficence towards humans vs. non‐malfeasance towards animals), Bentham's 1789 utilitarianism (Bentham 2007) (acceptable if adequate human benefit is expected), mid‐20th century animal rights (acknowledge animals as having intrinsic rights, on a varying scale relative to humans), respect/dignity (Anderson and Perry 1999) as per “the code” 1978 (acknowledge animals as respectable entities, on a varying scale relative to humans), the Dutch 1981 legislated inherent value (Brom and Schroten 1993) (acknowledge animals as having intrinsic value, on a varying scale relative to humans), Francione's 1996 abolitionism (Francione 2010) (stopping animal research completely), Garner's 2013 justice (“morally fair” treatment of animals), Kantian‐derived “fellow creatures” (acknowledge animals as our counterparts in sharing the world), etc., are elaborately debated, but out of scope of this study (Brom 2002).

Animal research ethics may also be placed in the wider context of the human use of animals. It is interesting to observe the 1986 Council of Europe Convention ETS No. 123 preamble (Council of Europe, 1986) making this philosophical construct systematically as follows:

“The member States of the Council of Europe, signatory hereto,…..

- Recognising that man has a moral obligation to respect all animals and to have due consideration for their capacity for suffering and memory;
- Accepting nevertheless that man in his quest for knowledge, health and safety has a need to use animals where there is a reasonable expectation that the result will be to extend knowledge or be to the overall benefit of man or animal, just as he uses them for food, clothing and as beasts of burden;
- Resolved to limit the use of animals for experimental and other scientific purposes, with the aim of replacing such use wherever practical, in particular by seeking alternative measures and encouraging the use of these alternative measures;
- Desirous to adopt common provisions in order to protect animals used in those procedures which may possibly cause pain, suffering, distress or lasting harm and to ensure that where unavoidable they shall be kept to a minimum, Have agreed as follows:”

Russell's and Burch's ***replacement*** principle (first R) involves “‘non‐sentient’ material which may in the history of experimentation replace methods which use ‘conscious living vertebrates’” (Russell and Burch 1959). It entails relative (substitution) or absolute (no animals) replacement. It is to be noted that animal experimentation is not restricted to “more sentient” vertebrates alone. It clearly extends to “relatively less sentient” invertebrates as well. Three important invertebrate species that have contributed substantially to the areas of cell biology and genetics are the nematode *Caenorhabditis elegans*, bakers's yeast Saccharomyces cerevisiae, and the arthropod *Drosophila melanogaster* (fruit fly). An excellent workshop report by Kretlow et al. (2010) summarises the significant role of these three invertebrates in biomedical research – the nematode *C. elegans* (apoptosis, RNA interference, developmental genetics), baker's yeast *S. cerevisiae* (genome sequencing, ageing, mitochondrial diseases), and the fruit fly *D. melanogaster* (genetic modelling, developmental biology, transformation, mutation, toxicity screening). In our own area of research pertaining to *Pseudomonas aeruginosa* pathogenesis, the nematode *C. elegans* has been used instead of mice models, the standard model for pathology and mortality studies (Tan et al. 1999).

***Reduction*** (second R) entails the balance of statistically significant numbers and minimising the number of animals. The “humaneness”(Russell and Burch 1959) of decreasing the animal numbers required for a study is quite obvious. Researchers utilising animals in their research have to substantiate how they conform both to the minimum statistically significant animal numbers needed for the study (power analysis), and to the principle of reduction. This information has to be made available both during research grant funding applications, and during animal ethics approval applications.

The third R, ***refinement***, presents more of a challenge, owing to its diverse possibilities, in innumerable scenarios. Refinement is so flexible that a unique refinement may be possible in each study, every time. Refinement “might be regarded as an art or an ability to improvise” (Russell and Burch 1959). Morton (1998) defines refinement as, “those methods which avoid, alleviate or minimise the potential pain, distress or other adverse effects suffered by the animals involved, or which enhance animal wellbeing.” Moreover, refinement not only attempts to reduce negative states in animals, but promotes “positive mental and physical states.”

## Mandatory Institutional Ethical Reviewing of Animal Experimentation: Chronological Sequence of Pioneering Nations

The second intent of our study is to demonstrate how previous and contemporary thought on principles/mores pertaining to animal experimentation ethics, finally translated into concrete action, via the implementation of concrete legislation to mandate compulsory review of ethical practices in animal research.

The goals and strengths of institutional animal ethical review include setting guidelines for ascertaining whether animals are necessary for a study in the first place, overseeing humane experimental conduct of animal studies, ensuring species‐specific tailoring of methodology, minimising biological variability, and establishing minimum discomfort to experimental animals. To start with, mandatory ethical requirements are needed to ascertain whether animals are needed for a particular study in the first place. The projected scientific outcome and impact must be weighed against the wellbeing of the animals to be used. This justification must include evaluation of the maximum number of study parameters that have the potential to impact negatively on the animal's wellbeing, an assessment of the knowledgeability of the investigators regarding these parameters, and their ability of remediation of the same. Variability in the choice of research animals reduces the detectability power of a study, and increases the number of animals required for statistical reliability. Contrariwise, variability per se may be essential to the study in question, as in certain toxicology studies (Biggers et al. 1958). Ethical reviewing must scrutinise these elements to ensure observance of the 3Rs. The ethics review is also needed to confirm that putative animal‐handlers are familiar with species‐specific indicators of distress, pain, disease, and abnormal behaviour. Additionally, the animal‐handlers must be endowed with the ability to assist in obviation of the same.

There are several potential weaknesses and drawbacks of institutional animal ethical review. Institutional animal ethical review may or may not have teeth, depending on the local environment. Legislation conflicts or common law precedents may dilute penalties in case of infringements of ethics. How “severe” would the punishment have to be? Suspension of animal experimentation may just be temporary in some cases. How does a body rehabilitate a “repeat offender”? There may be doubtful efficacy in the monitoring of procedural consistency. It would be difficult to oversee data and indicators from a plethora of working individuals under the ambit of an ethics committee review approval. The interinstitutional and intrainstitutional variability between the qualities of animal ethics reviewing will be difficult to ascertain. Therefore, quality control and consistency will always be in doubt.

In developed countries, the pioneering nations which were most concerned about adherence to animal experimentation ethics, systematically and progressively mulled ideas, discussed them in the legislature, and finally promulgated mandatory institutional ethical reviewing of animal experimentation. The chronology of these events varied from country to country. In general, it took a few decades after initially contemplating the ideas, to finally implementing laws pertaining to mandatory institutional ethical reviewing of animal experimentation. Developing countries generally do not have laws mandating institutional ethical reviewing of animal research. However, a few developing countries like India, regardless of adherence or non‐adherence, have introduced legislation similar to their Western counterparts.

The forerunning nations or jurisdictions which pioneered mandatory institutional ethical reviewing of animal experimentation are as follows.

### Australia (1978)

Animal experimentation has been governed by the “Australian Code of Practice for the Care and Use of Animals for Scientific Purposes” (the Code) since 1969 (Anderson and Perry 1999). However, the requirement of institutions to establish Animal Experimentation Ethics Review Committees (scientists and non‐scientists) was indicated only in the 2nd edition of this Code (1978) (Ewing 1989). Further details and functional itemisation were done in the Code's subsequent editions.

### Sweden (1979)

After a 3‐year pilot program, animal ethics committees (six regional committees) were made compulsory (1979) (Hagelin et al. 2003).

### Canada (1980)

In 1980, Canada mandated institutions to establish local animal care committees to review reviewing ethical features of animal experimentation protocols (CCAC, 1980). The autonomous Canadian Council on Animal Care (CCAC) advises and supervises the surveillance of animal care and experimentation in Canada's universities, government laboratories and pharmaceuticals (Rowsell 1986).

### The USA (1985 edition of 1963‐published “The Guide”)

The “Guide for the Care and Use of Laboratory Animals” (the Guide) was first published in 1963, under the title “Guide for Laboratory Animal Facilities and Care”, and was revised at least six times (Committee for the Update of the Guide for the Care and Use of Laboratory Animals, 2011). In the 1985 edition, the Guide mandated institutions to appoint committees (with one noninstitution affiliated member) to evaluate animal care and experimentation (Committee for the Update of the Guide for the Care and Use of Laboratory Animals, 2011).

### India (1998)

The 1998 Indian gazette notification (and its 2001 amended notification) by the Committee for the Control and Supervision of Experiments on Animals (CPCSEA) mandates local institutional animal ethics committees (Committee for the Purpose of Control and Supervision of Experiments on Animals, 1998).

### The UK (2000)

The United Kingdom (UK) Home Office ***made it compulsory (2000)*** for institutions to “introduce an animal experimentation ethical review process” (Home Office, 2014). Prior to this, the Cruelty to Animals Act 1876 (An Act to Amend the Law Relating to Cruelty to Animals) was supplanted by the Animals (Scientific Procedures) Act 1986 (ASPA) – ***both of them did not mandate*** a requirement for institutional ethical reviewing of animal use in research (Home Office, 2014). However, it is indeed noteworthy that the pioneering Cruelty to Animals Act 1876 first established a licensing system with a relative degree of prospective evaluation, in addition to the establishment of a monitoring inspectorate. Vestiges of the Cruelty to Animals Act 1876 (sans local animal ethical review) are still within the legal framework in a few erstwhile British colonies, but are out of scope of further elaboration herein.

### The EU (1986 & 2010) – Inclusive of the UK

The EU 86/609/EEC directive (1986) was issued by the European Union (EU) to promote ethical adherence in animal research (https://conventions.coe.int/Treaty/en/Treaties/Word/123.doc) (Council of Europe, 1986). The 2010/63/EU directive (2010) on the Protection of Animals Used for Scientific Purposes (http://eur-lex.europa.eu/legal-content/EN/TXT/?uri=celex:32010L0063)(European Commission 2010) makes holistic project evaluation (including harm‐benefit analysis) compulsory before conducting animal research. However, ***both the EU 86/609/EEC directive (1986) and the 2010/63/EU directive (2010) do not require institutional review by committees***. In 2006, the Federation of European Laboratory Animal Science Associations (FELASA) identified 16 EU countries (amongst the 20 EU countries reviewed by them) as having robust ethical reviewing as a compulsory prerequisite to conducting animal research (Smith et al. 2007). In their analysis, FELASA posited that “ethical review should aim to ensure that, at all stages in scientific work involving animals, there is adequate, clearly explained ethical justification for using animals” (Smith et al. 2007). Therein, FELASA not only emphasised the necessity of harm‐benefit analyses prior to embarking on research projects involving animals, but also underscored the importance of “normative” animal research ethical review processes to reflect diverse ethical perspectives (Smith et al. 2007).

There is great diversity of the “organisation” of ethical review processes for animal experimentation in developed countries. For example, please refer to Smith et al. (2007) for a concise summary (Table 1) of the wide range of ethical review processes organisation of laboratory animal use in the European Federation of Laboratory Animal Science Associations (FELASA). We have chosen a few aspects (FELASA countries and elsewhere) which we found interesting and summarised them below as points.

**Table 1:** An overview of the organisation of ethical review of laboratory animal use in the Federation of Laboratory Animal Science Associations (FELASA) countries in Europe (Smith et al. 2007)

Country	Mandatory processes^a^	Voluntary processes
Austria	For academic institutions: **National committee** of the Ministry of Education, Science and Culture. Industry: **Official veterinarian**	Institutional committees in some facilities
Belgium	**Institutional committees** (which can be shared between institutions) and **Government inspectors** (who are members of the local committees) and a **National committee** when difficult issues arise	
Czech Republic	**Institutional committees**; two **National committees:** representing (i) all Ministries involved in animal experiments and (ii) the Academy of Sciences; final authorisation by a **Government committee**, the Central Commission for Animal Welfare and the Environment	
Denmark	Review by **National committee** appointed by the Minister of Justice which directs a **Government inspectorate**	Four institutional committees
Estonia	A **National licensing committee** was established at the Estonian Ministry of Agriculture in May 2004. The committee reviews applications and grants permits for animal experiments; meetings take place according to the number of applications received	
Finland	At the time of writing, institutional committees (some are shared between institutions). Changing to a **National Committee** as a result of a change in the law in 2006	
France	*Applications for licences are approved and given by the Ministry of Agriculture. Government veterinary inspectors from the local Veterinary Service in each Prefecture check compliance (field of research, training and competence of researchers). Painful protocols must be declared to the local Prefecture, and an additional licence and evaluation is required for use of non‐domestic animals. A **National Ethical Committee** oversees the good functioning of the ethical committees (but there is not as yet a legal requirement for researchers to submit their work for ethical review by these committees)*	Regional committees for public research (22); Institutional committee in each industrial firm^b^
Germany	Review by **institutional Animal Welfare Officer** (a veterinarian, medical doctor or zoologist), then by **Regional committee** (c. 40) advising the government authorities	
Greece	**Official veterinarian** from the Local Veterinary Service in each Prefecture, who may take advice from scientists in the relevant field of work	Institutional committees in Medical Faculties and some research institutions
Ireland	*Applications for licences must be approved by the **Minister for Health and Children**. A local **nominated competent person** (preferably a veterinary surgeon) must review each application and declare that he/she does not envisage any practical difficulties on welfare grounds and specify any reservations*	Institutional committees in most institutions
Italy	*A review by a special **Commission at the National Institute of Health** is required only for: procedures involving cats, dogs, non‐human primates and/or endangered species; procedures without anaesthesia; and those for education and training*	Institutional committees in most research centres
Latvia	**National committee**, at the Latvian Council of Science	
Lithuania	**National committee** of the State Food and Veterinary Service	Institutional committees in some facilities
Netherlands	**Local (mostly institutional) committees**, plus a **National committee** which acts as a ‘court of appeal’ when a local committee has rejected a proposal (very rare). The law permits the outsourcing of ethical review, so that ‘institutional’ committees can advise more than one institution, and there can also be independent committees (there is one at present), whose services can be hired by institutions do not have their own	
Norway	**Local ‘competent person’** and **National committee** (National Animal Research Authority – for review of cases which the local competent person finds too controversial to make a decision, or is involved in, field experiments, and painful experiments where painkillers are withheld (very rare))	Institutional committees in some facilities
	A new Animal Welfare Act is currently being drafted	
Poland	**Regional committees** (18) set up by the **National Ethics Committee on Animal Experimentation (NEC/AE)** which oversees their work as an appeal authority.	
Spain	***Regional committees** in Catalonia, Andalusia and Aragon; i**nstitutional committees** in all research centres in Catalonia and Aragon. From October 2005, a new national law requires **institutional committees** in all State (but not other) research centres, and sets up a **State Ethical Commission of Animal Welfare** which must approve and supervise high severity procedures*	Institutional committees in most other research centres in the remaining regions
Sweden	**Regional committees** (7)	
Switzerland	**Regional committees** (10), which advise the Cantonal Authority whether or not experiments should be authorised; plus a **National committee** to advise the cantons in controversial cases and more general matters. The **Federal Veterinary Office** has the right to appeal.	Institutional committees in some facilities
UK	**Institutional committees and other local processes** review project licence applications as well as more general matters pertaining to the care and use of laboratory animals within institutions. Applications then forwarded to **Government inspectors** who, having weighed the likely welfare costs against the potential benefits, advise the Secretary of State for the Home Office whether or not they should be granted. There is also a **National committee** (the Animal Procedures Committee) for general advice on the operation of the law and ethical review of certain classes of licence application	

a *Italics* indicate countries in which there is not yet a national, mandatory requirement for prior ethical review of all regulated scientific uses of animals

b Although not legally required, the organisations involved signed a binding commitment to submit work to these processes for ethical review.

This table summarises the wide range of general organisation of ethical review processeses of laboratory animal use in the Federation of Laboratory Animal Science Associations (FELASA) countries in Europe.(Smith et al. 2007) [License number to reproduce table from SAGE Publications ‐ 4115900032136]

1. There is a high degree of consensus (predominantly amongst developed countries) on animal research ethics, harm‐benefit analysis (e.g., Bateson's decision cube with three research dimensions – quality, suffering, and benefit), and the necessity for systematic animal research ethical review processes. This was elaborately analysed and described by the International Council for Laboratory Animal Science (ICLAS) in 2010.(ICLAS – International Council for Laboratory Animal Science, 2010)
2. A country may have mandatory ethical evaluation by a person (chosen from selected and endorsed individuals), but not by a committee (e.g., Norway) (Smith et al. 2007).
3. A country may have national ethical review bodies feature legislated, without mandating local institutional bodies.
4. A country may have national or professional body codes/guidelines, but animal experimentation legislation may or may not reference them.
5. Legislation and regulations are not synonymous, although regulations generally stem from legislation.
6. Legislation may be delegated regionally (e.g., Australia and Canada), but the regional law may or may not include enforcement of the code of guidelines (White 2007).

Research pertaining to the efficacy of institutional ethical reviewing of animal research is sparse. We speculate that institutional ethical reviewing may work better in countries (and circumstances) which are more developed, have better funding for animal facilities, have lesser bureaucratic impediments, have simpler/more direct processes, and have flexible common/statutory law providing allowance for better reviewing and penalty implementation. Institutional ethical reviewing may not work as well in countries (and circumstances) with the opposite of what was just mentioned.

## Teaching ethics in animal experimentation pertaining to human disease remediation

It is indeed important to note that not only generally, but also specifically in our discipline; animal research per se is rather distinct from animal research teaching. Our biomedical/and pathophysiological research aims to judiciously explore and garner hitherto unknown knowledge. However, animal research (and ethics) teaching aims to illustrate already known facts. This has fundamental implications for the ethical reviewing of animal use, and for the necessity of justifying the indispensability of animal use. Pertaining to teaching animal research ethics, we have found widespread conceptual similarities in basic content of animal research ethics courses across multiple Sydney‐based universities. However, the teaching styles and interactional content differ.

The third intent of our study, therefore, is to lay out suggestions, practical recommendations, and teaching strategies for the lucid inculcation of animal experimentation ethics to interested parties, namely scientists, students, and clinicians. This is an extremely important intent, owing to the necessity for clarity in the transfer of information, mores, and legalities.

### Recommended and/or familiar components of a typical teaching course on animal experimentation ethics

Teaching ethics in animal experimentation pertaining to human disease remediation ought to be concise, but empathetic. Audiovisuals will certainly make the teaching much more interesting. A typical animal experimentation ethics course at an institution or university should topically involve the following, or segments of it thereof.

1. Evolution of thinking in animal ethics
  - Recognise that animal experimentation ethics is to be placed in a wider context of the human use of animals (Council of Europe, 1986), and ethics as depicted in the chapter by Olssson, et al. in the 2010 edition of the Handbook of Laboratory Animal Science (Olsson 2010).
  - History of the local institutional animal ethics committee, and relevant legislation (local and international)
2. Russell's and Burch's 3R principles (Russell and Burch 1959)
3. A scientist's ethical responsibilities
  - Recognition and relief of distress and pain in experimental animals
4. Summary of extant animal models
  - Well‐established alternatives to animal experimentation
  - Emerging alternatives to animal experimentation
5. Strategising animal experiments with careful heed to Russell's and Burch's 3R principles (Russell and Burch 1959)
6. Tips on writing proposal submissions to the local animal ethics committee, with emphases on the following:
  - Clear objective(s)
  - Targeting non‐scientists – Where? What? Why? How? Who? When?
  - Clarity in thought process, and sequence of experiments
  - Concise and appropriate literature summary
  - Animal number estimates – minimisation and justification
  - Personnel involvement – stratification, description, and justification
  - Stage of study – pilot experiments? Transitional stage? Main study corpus?
  - Record keeping strategies/commitments, and accountability hierarchy
  - Interventional techniques, details of surgery, pain/distress‐management
  - Candid and crystal‐clear endpoint(s)
7. Animal welfare scrutiny and monitoring
  - Animal experiment record keeping, mishap reporting/redressal, and feedback addressal
8. Short lecture on research procedures
  - Few demonstration sessions and lectures on animal handling, and common techniques (anaesthetisation, blood sampling, euthanasia simulation, etc.)
9. Reading material (“homework – whole/links or piecemeal/bibliography”)
  - Relevant national legislation on animal experimentation ethics
  - The authorised national code of ethics on animal experimentation
    - For example, the Australian Code (Ewing 1989; Anderson and Perry 1999) section on justification posits thus: “Projects using animals may be performed only after a decision has been made that they are justified, weighing the predicted scientific or educational value of the projects against the potential effects on the welfare of the animals”.
  - Guidelines to common animal experimentation procedures
  - Guidelines to promote experimental animal well‐being

### Ideal learning endpoints

Utilising a multiple‐choice test at the end of a course, the course participants would be assessed for a “reasonable” comprehension of (percentile scores or percentage cut‐offs):

1. The spectrum of ethical issues pertaining to animal experimentation
2. A scientist's ethical responsibilities
3. A practical application of Russell's and Burch's 3R principles (Russell and Burch 1959)
4. Application submission procedure to the local animal ethics committee
5. Recognition and relief of distress and pain in experimental animals
6. Basic animal handling, anesthetisation, blood collection, drug administration, and euthanasia

We consider these components and endpoints to be essential as these topics adequately cover the legal, administrative, ethical, statistical (basic), and technical (basic) aspects of animal experimentation.

## Emerging alternatives to animal experimentation pertaining to human disease remediation

The fourth and last intent of our study is to provide available alternatives to animal research to raise awareness of viable, and at times, even better options outside of animal experimentation. This would directly and indirectly feedback on our first intent extension of encouraging relevant personnel to better conform to these ethical standards.

Outside of the well‐established alternatives to animal experimentation like tissue culture methods including primary/continuous/immortalised cell lines, explant cultures, organ cultures, several recent strategies have been recently mooted to curtail animal experimentation, and simultaneously (and surprisingly) improve efficacy of data‐gathering. We used Pubmed searches (using replacement, in vitro alternatives, human tissue, etc., as search words) to identify those alternative methods which we thought would have “maximum impact” (well‐cited research or high‐impact journals on this subject or articles associated with our reseach).

While alternatives to animal experimentation may reduce research dependence on animal (through replacement); they currently cannot replace animal testing altogether. This impossibility exists despite several ethical, political, and financial “incentives” to persevere in this direction. The extant alternatives serve to complement animal experimentation in current research.

### In vitro models

#### Organs on chips

**Figure 1:**
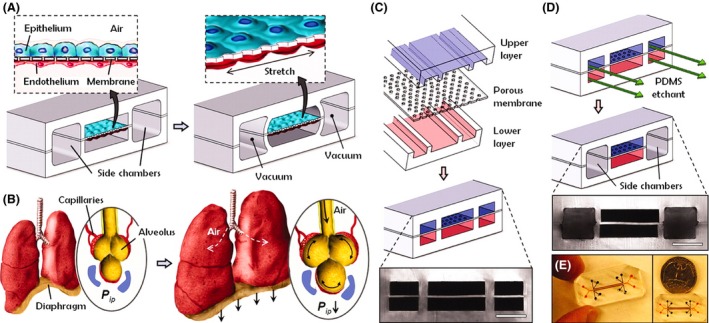
Organ on Chip – the human “lung‐on‐a‐chip” microsystem (Huh et al. 2010, 2013). (A) The microfabricated lung mimic device uses compartmentalised polydimethylsiloxane (PDMS) microchannels to form an alveolar‐capillary barrier on a thin, porous, flexible PDMS membrane coated with extracellular matrix (ECM) – fibronectin or collagen. The device recreates physiological breathing movements by applying vacuum to the side chambers and causing mechanical stretching of the PDMS membrane forming the alveolar–capillary barrier. (B) During inhalation in the living lung, contraction of the diaphragm causes a reduction in intrapleural pressure (*P*_ip_), leading to distension of the alveoli and physical stretching of the alveolar–capillary interface. (C) Three PDMS layers are aligned and irreversibly bonded to form two sets of three parallel microchannels separated by a 10‐*μ*m thick PDMS membrane containing an array of through‐holes with an effective diameter of 10 *μ*m. Scale bar, 200 *μ*m. (D) After permanent bonding, PDMS etchant is flowed through the side channels. Selective etching of the membrane layers in these channels produces two large side chambers to which vacuum is applied to cause mechanical stretching. Scale bar, 200 *μ*m. (E) Images of an actual lung‐on‐a‐chip microfluidic device viewed from above. [License number to reproduce image from The American Association for the Advancement of Science ‐ 4115900305591].

The most interesting “animal substitute” to buttress preclinical drug development is the Organs on chips (OOC), pioneered by scientists from Harvard University and University of Pennsylvania (Huh et al. 2010). Microfabrication methodology from the computer microchip manufacturers was utilised to devise microengineered systems capable of supporting living cells. The OOC looks promising as a pathophysiologically pertinent model of experimentation. OOCs are “micro‐engineered biomimetic systems containing microfluidic channels lined by living human cells, which replicate key functional units of living organs to reconstitute integrated human organ‐level pathophysiology in vitro” (Huh et al. 2013). A seminal protocols paper (Huh et al. 2013) describes the first OOC innovation. This pioneering model describes not only how a “‘breathing, elastic’ lung‐on‐a‐chip” (Fig. 1) is crafted, but also how their protocol can be modified to develop other human organ chips, like a ‘peristaltic’ gut‐on‐a‐chip’”.

#### Human‐derived three‐dimensional tissue models: Epidermis–dermis human skin equivalents

In vitro models of skin pathophysiology and drug testing has been around for some time. Pioneering testing of human skin equivalents (HSE) included EpiDerm (Monteiro‐Riviere et al. 1997) and full‐thickness EpiDerm (Kubilus et al. 2004). Presently, HSE models include a huge spectrum, ranging from those used to demonstrate simple physiology, to those used to analyse model diseases (from autoimmune disorders to malignancies) (Auxenfans et al. 2009; Semlin et al. 2011). Contingent on standardisation and quality, these models may be better than animal models. This is partly because the originating skin samples are human‐derived. Additionally, these tissue models are grown in vitro in a biochemical and physiological milieu closely simulating human homeostatic conditions.

#### Human‐derived three‐dimensional tissue models: Others

Using Russel and Burch's principle of replacement (Russell and Burch 1959), several human‐derived three‐dimensional models have been synthesised, tested, validated (a few of the several), and used (Sheasgreen et al. 2009). These 3‐D in vitro models have not only an “ethical edge”, but also a more pathophysiologically relevant edge owing to the human origin of these tissues. This edge is obvious as the tissue samples originate from humans, and are grown in vitro in a homeostatic environment akin to human biochemical and physiological conditions. Additionally, an animal model (like a commonly used inbred murine model) has far less biological and genetic differences compared to the complex human genetic/biological heterogeneity, making human tissue much more physiologically relevant. These three‐dimensional human‐derived tissue models include oral epithelia (Klausner et al. 2007), gastrointestinal epithelia (Sheasgreen et al. 2009), vaginal epithelia (Ayehunie et al. 2011), ocular tissue (Kaluzhny et al., 2011), gingival tissue (Hai et al. 2006), respiratory epithelia (Sexton et al. 2011), and dendritic antigen‐presenting cells (Sheasgreen et al. 2009).

#### Human blood derivatives

The European Partnership for Alternative Approaches to Animal Testing (EPAA) (Cozigou et al. 2015) is a voluntary collaboration intending to focusing information and resources to the experimental use of animals in regulatory testing. The collaborating partners include the European Commission, individual companies from seven industrial sectors, and their European trade federations. The European Commission is bound by the EU Treaty to maximally promote 3R principles (Cozigou et al. 2015). The seven industrial sectors include the European Chemical Industry Council (Cefic), the European Federation of Pharmaceutical Industries and Associations (EFPIA), the European Cosmetic Toiletry and Perfumery Association (Colipa), the European Association for Bioindustries (EuropaBio), the International Federation for Animal Health Europe (IFAH‐Europe), the International Association for Soaps, Detergents and Maintenance Products (A.I.S.E), and the European Crop Protection Association (ECPA). The EPAA indicated the necessity to include authorities during the validation of, and the legal acceptance processes pertaining to animal experimentation alternatives (Montag et al. 2007). The pyrogen complement activation test (human plasma complement activation test) (Sladowski et al. 2001), the alternative pyrogen test (monocyte activation test) (Schindler et al. 2009), and the whole blood cytokine‐release immunotoxicity test (Langezaal et al. 2002) are three such processes investigated under EPAA's auspices. These tests display enormous potential to replace the limulus amebocyte lysate (LAL) assay which requires a quarter of a million horseshoe crabs to be exsanguinated every year (30% of blood collected per animal) with a 3–15% post‐exsanguination mortality rate (Anderson et al. 2013).

Pathophysiologically relevant in vitro models serve as exemplary models of replacement and cost‐cutting, but replacement herein will still be partial, albeit significant. Animal testing will still be required for the foreseeable future. For example, in our research, a bacterial toxin had effects which were different from that on cultured cells (Cheluvappa et al. 2007c), than its in vivo effects in a live animal (Cheluvappa et al. 2008). Similarly a tested drug, owing to a multitude of reasons, may work fine on an in vitro model, but may not work (or may work differently) on a live animal. Therefore, in vitro models will effectuate manifold prescreening processes prior to animal experimentation, but may only serve partially in reduction. Furthermore, only in vivo animal models can account for complex and/or unknown biological systems and pathways that in vitro models cannot encompass.

### Computer modelling – in silico and quantitative structure–activity relationship analyses

Pathophysiological simulations can now be screened using high‐tech computer modelling programs (in silico modelling) (Martonen et al. 2003; Aguda et al. 2011). Toxicity screening (Golbamaki et al. 2014) and fundamental pharmacokinetic events such as gut absorption, protein‐binding, endothelial barrier passage, etc. can also be done rapidly in vitro depending on specific in silico modelling program availability (Raunio et al. 2004). There are addition software‐based techniques (quantitative structure‐activity relationships or QSARs) (van Leeuwen et al. 2009) that utilise sophisticated estimates of a molecule's hazard‐inducing capacity, based on its similarity to existing molecules, and extant human physiology. QSAR software (toolboxes) (van Leeuwen et al. 2009) have been used extensively, either exclusively, or in conjunction with reduced animal numbers. Examples include hydrogenated azoles (Craig et al. 2014), sulphur‐containing compounds (Richarz et al. 2014), pesticide/biocide carcinogenicity (Devillers et al. 2011), unsaturated aliphatic aldehydes (Devillers and Mombelli 2010b), aromatic amines (Devillers and Mombelli 2010a), and chemical carcinogens (Mombelli and Devillers 2010).

Computer modelling is limited in its role in limiting animal use in research. To start with, animal research is essential to glean pathophysiological nuances, even before one starts to play with the keyboards. While available data may be used for extant in silico models, incorporation of future data, which may ostensibly be more complex, may necessitate further animal research. Processor speed and configuration adaptability are essential not only for designing intricate simulations, but also for using them (Zaslavsky et al. 2014). Such simulations generally focus on major aspects, and tend to overlook smaller, but equally (if not more) important aspects. Therefore, computer modelling may assist in preliminary vetting surveys ahead of more concrete experiments involving other models (including animals). This may partially assist with the reduction objective, the magnitude of which may fortunately or unfortunately depend on “research and funding priorities/popularity.”

### Research involving human volunteers

Positron emission tomography (PET) and functional magnetic resonance imaging (fMRI) pertaining to brain activity are the first approaches that come to mind when the topic of research involving human volunteers is broached. However, there are several other “human testing” investigative methods which have been used. A classic example is microdosing, the research pertaining to which, was first published a decade ago. Microdosing is “an approach to early drug development where exploratory pharmacokinetic data (with or without imaging) are acquired in humans using inherently safe sub‐pharmacologic doses of drug” (Lappin et al. 2013). It is essential to note that human ethics approval must be obtained prior to any microdosing experiment. In the US, institutional review boards (IRBs) come into play with regard to this. The human ethics review committees (HRECs) are the Australian equivalent of the US IRBs. It is essential (and obvious) that microdosing studies ought to be conducted on human volunteers without coercion. A large number of drugs (Lappin et al. 2013) have been investigated using microdosing, and around 80% of microdose pharmacokinetics published is commensurate with those observed at therapeutic doses, within a twofold difference. It is to be emphasised that the options for human testing have their own ethical and legal hurdles. They are not simple or simplistic substitutions for animal models. However, owing to the voluntary and cognizant nature of the test human subjects, a significant proportion of these hurdles are easily overcome. Microdosing may not have produced “concrete data” yet, but surely has much more to offer, being an excellent ***contributing tool***.

While the thought of human PET and MRI scans “lighting up” when activated under certain sensory/motor conditions sounds like an appealing example of replacement, we currently still require animals to devise and test the efficacy and safety of therapeutic approaches as in mortality or toxicity studies. On the other hand, microdosing inherently cannot predict adverse reactions of drugs that may occur at therapeutic levels, which animal studies clearly can. Therefore, microdosing can only assist in partial reduction of animal use in research.

## Conclusions

Animals have been used in research as it is generally purported to simulate human biology. The ethics pertaining to animal research evolved over centuries of philosophical traditions, and not rigid rules of operation, but an avenue to express our moral obligations towards research animals. Russell and Burch set of 3Rs (Replacement, Reduction, and Refinement) are currently the most utilised set of animal ethics. The countries which were most concerned about adherence to animal experimentation ethics finally promulgated mandatory institutional ethical reviewing of animal experimentation. The chronological sequence (in its pioneering developed nations) of national legislation in developed countries pertaining to mandatory institutional ethical reviewing of animal experimentation has been charted out in this work. Although developing countries generally do not have laws requiring institutional ethical reviewing of animal research, a few like India have introduced relevant legislation. While teaching animal research ethics, we must include topics pertaining to the legal, administrative, ethical, statistical (basic), and technical (basic) aspects of animal experimentation. In this work, our rendering of the fundamentals of teaching animal research ethics in institutions is discussed. We have laid out suggestions, practical recommendations, and teaching strategies for the lucid inculcation of animal experimentation ethics to scientists, students, and clinicians. In addition to “traditional” alternatives to animal experimentation (like tissue cultures), several innovations have been recently introduced with the objective to retrench animal experimentation. They include organs on chips, human‐derived three‐dimensional tissue models, human blood derivates, microdosing, and computer modelling. However, these alternatives can only reduce research dependence on animals (through replacement and reduction) by complementing animal research. We have a fair way to go!

## Author Contribution

Rajkumar Cheluvappa wrote and submitted the manuscript. Paul Scowen assisted in animal research ethics. Rajaraman Eri proposed the idea and wrote the manuscript.

## Disclosure

None.
